# Supplementary material for: Profile of congenital heart disease in infants born following exposure to preeclampsia
Source: PLoS One. 2020 Mar 26;15(3):e0229987. doi: 10.1371/journal.pone.0229987 (PMC7098553; doi:10.1371/journal.pone.0229987)
Supplement: Attachment — Submitted filename: Reviewer Notes.pdf [file pone.0229987.s003.pdf]

Reviewer Notes

PROFILE OF CONGENITAL HEART DISEASE IN INFANTS BORN FOLLOWING EXPOSURE TO PREECLAMPSIA

Summary:

This cohort study investigated the association between CHD in offspring of women with PE/E. They took into account for newborn sex, gestational age, and maternal factors (weight, age, and education). They reported that newborns from PE/E pregnancies were associated with an approximate 8 fold increase in risk for CHD. Most common of these defects included ASD, PFO, VSD, and PDA. Most of the PDA cases were resolved by the evaluation at day 28. These findings of this report is consistent with findings from other reports in observing increased CHD and decreased birth weight associated with PE/E pregnancies. However, this study re-evaluates the prevalence of PDA in infants from PE/E pregnancies. This study is unique in that it conducts a day 28 evaluation for CHD, therefore, accounting for PDA and PFO that may be transiently present in preterm infants. Overall, this paper provides statistical information about the association between PE/E pregnancies and increased CHD risk, and provides grounds for urging CHD screening for fetuses and newborns from PE/E pregnancies. Reviewers concerns are mentioned below with specific sections. The grammar throughout the paper needs to be reviewed with care.

Major Concerns:

1. In the abstract results section, you mention, “ASD (4 newborns), PDA (21 newborns), patent foramen ovale (14 newborns) and VSD (2 newborns) were the prevalent lesions in the first week of life”. The group (PE/E) to which this is referring to needs clarification.
2. In the abstract results section, you mention, “Being the infant of a woman with preeclampsia was associated with about 8-fold increased risk of having CHD (OR=7.9, 95%CI=2.5-24.9)”. If the p-value is mentioned it would provide more information about statistical significance.
3. In the introduction, you mentioned that one of the reasons to look into the association between CHD and PE/E is because PE/E itself entails an inflammatory state. However, before this statement is given, the etiology is not given. The reasoning could be more cohesive if a statement or two about PE/E and inflammation is provided in the first paragraph of this section.
4. In the results section, you mention “All of the PDA in newborns of normal pregnancy and 8 (8.9%) of those newborns of preeclamptic women had closed by the 28th day of life (Table 2)”. Table 2 shows that there are still 4 newborns (from PE/E) and 1 newborn (from normal pregnancy) with PDA. I believe it would be more accurate to say “most of the PDA cases” instead of “all”.
5. In the results section, you mention, “Being the infant of a woman with preeclampsia was associated with an ~8-fold increased likelihood of having CHD (OR=7.9, 95%CI=2.5-24.9). Being a female infant or being preterm was associated with an increased likelihood of having CHD of 1.8 times (OR=1.8, 95%CI=0.63-4.9) and 1.4

times (95% CI= 0.22-9.3) times respectively though not statistically significant". Including actual p-values when describing the statistics would backup your statement.

6. In the discussion section, you mention, "This risk is dose dependent and likely due to a shared angiogenic imbalance in the mother and fetus.20-22". What is the dose referring to? What is currently mentioned does not match the other statements or logic.

Minor Concerns:

1. Grammar errors throughout the paper. Please double check on the adverbs, verb tenses, subject verb agreement, conjunction usage, comma usage (often missing), and subject association.
2. Were the maternal nutritional status during pregnancy accounted for? Maternal nutrient status of various vitamins and minerals have a profound effect on fetal development.
3. It might be more informative to report the maternal weight in the form of BMI to account for their height.

PLOS One Questions:

1. What are the main claims of the paper and how significant are they for the discipline? <u>Answer: Mentioned is summary (above).</u>
2. Are the claims properly placed in the context of the previous literature? Have the authors treated the literature fairly? <u>Answer: The connection between inflammation and PE/E in the introduction section needs to be elaborated. This study could prompt further investigation on the link between PE/E and CHD if the authors elaborate on potential mechanisms of angiogenic imbalance that leads to CHD development.</u>
3. Do the data and analyses fully support the claims? If not, what other evidence is required? <u>Answer: Yes, all of their claims are based on observed results and statistics.</u>
4. *PLOS ONE* encourages authors to publish detailed protocols and algorithms as supporting information online. Do any particular methods used in the manuscript warrant such treatment? If a protocol is already provided, for example for a randomized controlled trial, are there any important deviations from it? If so, have the authors explained adequately why the deviations occurred? <u>Answer: They utilized standardized diagnostic protocols for CHD. Statistical analysis methods are also provided.</u>
5. If the paper is considered unsuitable for publication in its present form, does the study itself show sufficient potential that the authors should be encouraged to resubmit a revised version? <u>Answer: This report needs major grammar editing before final submission.</u>
6. Are original data deposited in appropriate repositories and accession/version numbers provided for genes, proteins, mutants, diseases, etc.? <u>Answer: The author declares that all data collected from the study is made available to the public.</u>
7. Are details of the methodology sufficient to allow the experiments to be reproduced? <u>Answer: Yes, sufficient methods are provided.</u>

8. Is the manuscript well organized and written clearly enough to be accessible to non-specialists? Answer: Yes, very clear organization. However, needs major grammar editing for smooth reading.
