# Supplementary material for: Profile of congenital heart disease in infants born following exposure to preeclampsia
Source: PLoS One. 2020 Mar 26;15(3):e0229987. doi: 10.1371/journal.pone.0229987 (PMC7098553; doi:10.1371/journal.pone.0229987)
Supplement: Attachment — Submitted filename: Response_Reviewers.doc [file pone.0229987.s004.doc]

Department of Paediatrics

University of Jos

PMB 2084,

Jos

Nigeria

23^rd^ January 2020

Dear Editor,

Thank you for the opportunity given to submit our manuscript for review. We also thank the reviewers for taking time to read, critique and review our manuscript. We also appreciate their useful comments and suggestions.

Find below our response to the Reviewers.

Reviewer number 1

1. Table 2, in preeclampsia group, the total number of PDA is 4, however if you counted the sum of three category (isolated, with PFO and ASD), the number is just 2 (just in with ASD). What is the problem? Is there a mistake?

**Response:**

**The total number of PDA is 4 (2 with isolated lesions, 2 associated with ASD). It was a clerical error.**
2. In the discussion, authors said,” Though prematurity is associated with a higher prevalence of CHD globally, our study did not find a significant association between prematurity and the odds of having CHD.” How can they get this opinion? Because in table 1 we can see that mean gestational age of women with preeclampsia or normal pregnancy is significantly different. Neonates whose mother with preeclampsia are premature and have a high risk of CHD. So, I think authors cannot give this conclusion.

**Response:**

**Even though women with Preeclampsia have a significantly lower gestational age, our results in Table 3 showed no significant association between prematurity and risk of CHD. This is why we made the statement above.**
3. As the design of this study, have all the mothers been excluded the genetic disease of heart, have they all detected the TORCHES. How about the history of delivery? Will it affect the health of newborns? Like the age of the first pregnancy and times of pregnancy and delivery.

**Response:**

**None of the women had a clinical history of TORCHES. However, because of the peculiarity of our developing economy, pregnant women do not have genetic studies done routinely. In addition, the scope of our study did not include genetic study. Timing of delivery, mode of delivery and parity of the women was not significantly associated nor did it differ between the two cohorts. But we will in future look in that direction.**

**Reviewer 2**

Major Concerns:
1. In the abstract results section, you mention, “ASD (4 newborns), PDA (21 newborns), patent foramen ovale (14 newborns) and VSD (2 newborns) were the prevalent lesions in the first week of life”. The group (PE/E) to which this is referring to needs clarification.

**Response:**

**This statement makes reference to the overall spectrum of CHD in the total population and not in women with Preeclampsia. The statement about PE/E ends with the previous sentence.**

2. In the abstract results section, you mention, “Being the infant of a woman with preeclampsia was associated with about 8-fold increased risk of having CHD (OR=7.9, 95%CI=2.5-24.9)”. If the p-value is mentioned it would provide more information about statistical significance.

**Response:**

**Noted and corrected**
3. In the introduction, you mentioned that one of the reasons to look into the association between CHD and PE/E is because PE/E itself entails an inflammatory state. However, before this statement is given, the etiology is not given. The reasoning could be more cohesive if a statement or two about PE/E and inflammation is provided in the first paragraph of this section.

**Response:**

**Noted and corrected.**

4. In the results section, you mention “All of the PDA in newborns of normal pregnancy and 8 (8.9%) of those newborns of preeclamptic women had closed by the 28th day of life (Table 2)”. Table 2 shows that there are still 4 newborns (from PE/E) and 1 newborn (from normal pregnancy) with PDA. I believe it would be more accurate to say “most of the PDA cases” instead of “all”.

**Response:**

**Noted and corrected to read “**The PDA in newborn….”
5. In the results section, you mention, “Being the infant of a woman with preeclampsia was associated with an ~8-fold increased likelihood of having CHD (OR=7.9, 95%CI=2.5-24.9). Being a female infant or being preterm was associated with an increased likelihood of having CHD of 1.8 times (OR=1.8, 95%CI=0.63-4.9) and 1.4 times (95% CI= 0.22-9.3) times respectively though not statistically significant”. Including actual p-values when describing the statistics would backup your statement.

**Response:**

**Noted and corrected.**
In the discussion section, you mention, “This risk is dose dependent and likely due to a shared angiogenic imbalance in the mother and fetus.20-22”. What is the dose referring to? What is currently mentioned does not match the other statements or logic.

**Response:**

**The statement is hereby deleted.**

Minor Concerns:
1. Grammar errors throughout the paper. Please double check on the adverbs, verb tenses, subject verb agreement, conjunction usage, comma usage (often missing), and subject association.

**Response:**

**Noted with thanks. Will correct that.**
2. Were the maternal nutritional status during pregnancy accounted for? Maternal nutrient status of various vitamins and minerals have a profound effect on fetal development.
It might be more informative to report the maternal weight in the form of BMI to account for their height.

**Response:**

**Maternal nutritional status assay of vitamins and other nutrients was not done.**

Once again, we appreciate the opportunity given to us to submit our manuscript. We also appreciate the great work done by the reviewers in reviewing our manuscript.

Yours faithfully,

Christopher Yilgwan
