# Supplementary material for: Profile of congenital heart disease in infants born following exposure to preeclampsia
Source: PLoS One. 2020 Mar 26;15(3):e0229987. doi: 10.1371/journal.pone.0229987 (PMC7098553; doi:10.1371/journal.pone.0229987)
Supplement: Attachment — Submitted filename: Reviewer 2. Second Review.docx [file pone.0229987.s005.docx]

Reviewer 2

Major Concerns:

1. In the abstract results section, you mention, “ASD (4 newborns), PDA (21 newborns), patent foramen ovale (14 newborns) and VSD (2 newborns) were the prevalent lesions in the first week of life”. The group (PE/E) to which this is referring to needs clarification.
   a. Response: This statement makes reference to the overall spectrum of CHD in the total population and not in women with Preeclampsia. The statement about PE/E ends with the previous sentence.

2^nd^ Review: I understand now. But may be helpful to clarify by saying, “ASD (4 newborns), PDA (21 newborns), patent foramen ovale (14 newborns) and VSD (2 newborns) were the prevalent lesions among newborns of women without preeclampsia in the first week of life.”

2. In the abstract results section, you mention, “Being the infant of a woman with preeclampsia was associated with about 8-fold increased risk of having CHD (OR=7.9, 95%CI=2.5-24.9)”. If the p-value is mentioned it would provide more information about statistical significance.
   a. Response: Noted and corrected

2^nd^ Review: Verified.

3. In the introduction, you mentioned that one of the reasons to look into the association between CHD and PE/E is because PE/E itself entails an inflammatory state. However, before this statement is given, the etiology is not given. The reasoning could be more cohesive if a statement or two about PE/E and inflammation is provided in the first paragraph of this section.
   a. Response: Noted and corrected.

2^nd^ Review: Verified. I think the current version provides a very detailed background and justification for the study.

4. In the results section, you mention “All of the PDA in newborns of normal pregnancy and 8 (8.9%) of those newborns of preeclamptic women had closed by the 28th day of life (Table 2)”. Table 2 shows that there are still 4 newborns (from PE/E) and 1 newborn (from normal pregnancy) with PDA. I believe it would be more accurate to say “most of the PDA cases” instead of “all”.
   a. Response: Noted and corrected to read “The PDA in newborn….”

2^nd^ Review: Verified.

5. In the results section, you mention, “Being the infant of a woman with preeclampsia was associated with an ~8-fold increased likelihood of having CHD (OR=7.9, 95%CI=2.5-24.9). Being a female infant or being preterm was associated with an increased likelihood of having CHD of 1.8 times (OR=1.8, 95%CI=0.63-4.9) and 1.4 times (95% CI= 0.22-9.3) times respectively though not statistically significant”. Including actual p-values when describing the statistics would backup your statement.
   a. Response: Noted and corrected.

2^nd^ Review: Verified.

6. In the discussion section, you mention, “This risk is dose dependent and likely due to a shared angiogenic imbalance in the mother and fetus.20-22”. What is the dose referring to? What is currently mentioned does not match the other statements or logic.
   a. Response: The statement is hereby deleted.

2^nd^ Review: This statement is still there. What do you mean by dose? “This risk is dose dependent and likely due to a shared angiogenic imbalance in the mother and fetus.20-22”. The sentence after that also seems to not flow well right after this statement. The statement itself sounds a little redundant. Can you make the last few sentences of this paragraph more concise?

Minor Concerns:

1. Grammar errors throughout the paper. Please double check on the adverbs, verb tenses, subject verb agreement, conjunction usage, comma usage (often missing), and subject association.
   a. Response: Noted with thanks. Will correct that.

2^nd^ Review: Did not notice any major grammar issues.

2. Were the maternal nutritional status during pregnancy accounted for? Maternal nutrient status of various vitamins and minerals have a profound effect on fetal development. It might be more informative to report the maternal weight in the form of BMI to account for their height.
   a. Response: Maternal nutritional status assay of vitamins and other nutrients was not done. Once again, we appreciate the opportunity given to us to submit our manuscript. We also appreciate the great work done by the reviewers in reviewing our manuscript.

2^nd^ Review: Reporting the weight status of the mothers could be more informative if the weight was reported as BMI if available.
